# Pulsed Electromagnetic Field Therapy Improves Osseous Consolidation after High Tibial Osteotomy in Elderly Patients—A Randomized, Placebo-Controlled, Double-Blind Trial

**DOI:** 10.3390/jcm8112008

**Published:** 2019-11-17

**Authors:** Patrick Ziegler, Andreas K. Nussler, Benjamin Wilbrand, Karsten Falldorf, Fabian Springer, Anne-Kristin Fentz, Georg Eschenburg, Andreas Ziegler, Ulrich Stöckle, Elke Maurer, Atesch Ateschrang, Steffen Schröter, Sabrina Ehnert

**Affiliations:** 1 Siegfried Weller Institute for Trauma Research, Department of Trauma and Reconstructive Surgery, BG Unfallklinik Tübingen, Eberhard Karls Universität Tübingen, Schnarrenbergstr. 95, D-72076 Tübingen, Germany; pziegler@bgu-tuebingen.de (P.Z.); benjamin.wilbrand@student.uni-tuebingen.de (B.W.); emaurer@bgu-tuebingen.de (E.M.); aateschrang@bgu-tuebingen.de (A.A.); sschroeter@bgu-tuebingen.de (S.S.); sabrina.ehnert@gmail.com (S.E.); 2 Sachtleben GmbH, Haus Spectrum am UKE, Martinistraße 64, D-20251 Hamburg, Germany; falldorf@citresearch.de (K.F.); a.fentz@web.de (A.-K.F.); eschenburg@citresearch.de (G.E.); 3 Department of Diagnostic and Interventional Radiology, University of Tübingen, Hoppe-Seyler-Str. 3, D-72076 Tübingen, Germany; fabian.springer@med.uni-tuebingen.de; 4 StatSol Lübeck, Moenring 2, D-23560 Lübeck, Germany; ziegler@statsol.de; 5 School of Mathematics, Statistics and Computer Science, University of KwaZulu-Natal, Pietermaritzburg, Scottsville 3209, South Africa; 6 Center for Musculoskeletal Surgery, Charité—University Medicine Berlin, Augustenburger Platz 1, D-13353 Berlin, Germany; ulrich.stoeckle@charite.de

**Keywords:** extremely low frequency pulsed electromagnetic field (ELF-PEMF) therapy, high tibial osteotomy (HTO), osseous consolidation, bone specific alkaline phosphatase (BAP), elderly

## Abstract

Extremely low-frequency pulsed electromagnetic field (ELF-PEMF) therapy is proposed to support bone healing after injuries and surgical procedures, being of special interest for elderly patients. This study aimed at investigating the effect of a specific ELF-PEMF, recently identified to support osteoblast function in vitro, on bone healing after high tibial osteotomy (HTO). Patients who underwent HTO were randomized to ELF-PEMF or placebo treatment, both applied by optically identical external devices 7 min per day for 30 days following surgery. Osseous consolidation was evaluated by post-surgical X-rays (7 and 14 weeks). Serum markers were quantified by ELISA. Data were compared by a two-sided t-test (α = 0.05). Device readouts showed excellent therapy compliance. Baseline parameters, including age, sex, body mass index, wedge height and blood cell count, were comparable between both groups. X-rays revealed faster osseous consolidation for ELF-PEMF compared to placebo treatment, which was significant in patients ≥50 years (∆_mean_ = 0.68%/week; *p* = 0.003). Findings are supported by post-surgically increased bone-specific alkaline phosphatase serum levels following ELF-PEMF, compared to placebo (∆_mean_ = 2.2 µg/L; *p* = 0.029) treatment. Adverse device effects were not reported. ELF-PEMF treatment showed a tendency to accelerate osseous consolidation after HTO. This effect was stronger and more significant for patients ≥50 years. This ELF-PEMF treatment might represent a promising adjunct to conventional therapy supporting osseous consolidation in elderly patients. Level of Evidence: I.

## 1. Introduction

Several factors, such as age, diabetes, smoking, or infections, affect osseous consolidation and patient outcome after orthopedic and trauma surgery [1,2,3,4]. Specifically, with aged bones and depleted calcium stores, elderly patients are at an increased risk for non-unions [5]. Therapies to support bone formation, especially in elderly patients, are therefore of great interest.

One potential technique to promote bone formation is the application of electric or electromagnetic fields (EMF). The association of osseous healing and electrical potentials was first described in the 1960s: transmission of mechanical stimuli into electrical impulses at the cellular level, called electromechanical coupling, is considered the key mechanism inducing bone formation after injuries [6]. Since then, various electric and electromagnetic fields (EMFs) have been used to treat chondral lesions, osseous non-unions, different kinds of fractures, osteotomies, osteoporosis, and chronic wounds [7,8,9,10,11,12,13,14]. The described EMFs range from frequencies of 5 Hz to 200 kHz, resulting in electrical fields between 1 and 100 mV/cm in the tissue [15]. Besides varying frequencies, the reported EMFs also differ in treatment time, waveform and amplitude, so there is no recommendation for clinical use. Despite the overall positive effects of EMFs on bone formation [15,16], small numbers of cases, inhomogeneous study cohorts, and lack of placebo controls and/or blinding limit the validity of the studies [17,18,19,20,21]. Therefore, this technology remains a niche application.

Based on current standards of evidence-based medicine, there is still a lack of clinical studies investigating the influence of extremely low-frequency pulsed EMFs (ELF-PEMF) in bone healing. Recently, a frequency-dependent effect of ELF-PEMF on human osteoblasts was shown, where 16 Hz showed the strongest effect both on viability/proliferation and osteogenic differentiation [22]. As a possible underlying mechanism, MAPKinase signaling induced by superoxide radicals and hydrogen peroxide was identified [22,23]. In the preceding in vitro studies, only 7 min of daily ELF-PEMF treatment was sufficient to stimulate osteoblast function. Compared to existing in vivo studies, with treatment durations of several hours daily [20,21], this short treatment duration is thought to increase compliance [24]. Based on these studies, it was proposed that this specific ELF-PEMF can improve bone formation in vivo.

To prove this hypothesis, the aim of this double-blind, randomized, placebo-controlled, mono-center study was to evaluate the effects of this specific ELF-PEMF on osseous consolidation and correlating serum markers in patients who received a high tibial osteotomy (HTO), representing a highly homogenous population, concerning the surgical procedure, postoperative rehabilitation and expected bone metabolism. Subgroup analyses are performed to identify the possible influence of sex, BMI, age, and wedge height, with stronger treatment effects expected in elderly patients.

## 2. Experimental Section

### 2.1. Study Registration

The study was registered (16 July 2015) at the German register for clinical studies (DRKS) with the study ID DRKS00008870 before inclusion of the first patient.

### 2.2. Ethics Statement

The study was conducted in accordance with the 1964 Declaration of Helsinki (Ethical Principles for Medical Research Involving Human Subjects) and was approved by the local ethics committee (No.: 337/2015BO1; 08.06.2015). Prior to inclusion, all patients gave their written informed consent to participate in the study.

### 2.3. Study Population and Procedure

Patients undergoing an open wedge HTO because of a varus deformity of their mechanical axis (Mikulicz line), combined with osteoarthritis and pain in the medial compartment, were included in this study.

Inclusion criteria were: agreement to participate in the study, minimum age of 45 years (minimum age was adjusted to 40 years), and an HTO with an opening gap of 5–10 mm.

Exclusion criteria were: patients with disorders in the bone metabolism (e.g., osteoporosis), medication known to affect bone metabolism, intra-surgical bone grafting, intra-operative hinge fractures, and previous surgeries of the proximal tibia, as an accumulation of these factors may delay fracture healing [3,4].

**Figure 1:**
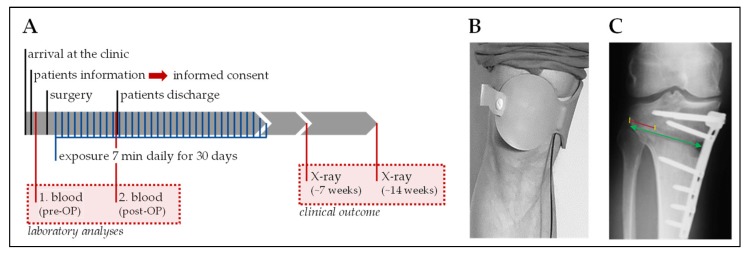
Overview of the study design. (**A**) Schematic overview of the study procedure. (**B**) Illustration of how the extremely low frequency pulsed electromagnetic field (ELF-PEMF) applicator (Somagen^®^ device) was placed during treatment. (**C**) Guideline for the quantification of osseous consolidation in high tibial osteotomy (HTO) patients [26]: the green arrow marks the osteotomy (distance A) and the red line marks the gap filling (distance B). Osseous consolidation is calculated as B/A × 100 (%).

Age, body mass index (BMI; kg/m^2^), nutritional status (NRS: Nutritional Risk Screening 2002 [25]), and pre-surgical blood values were recorded to assure homogeneity of the study population. Primary endpoints were speed and degree of osseous consolidation after 7 weeks and 3 months. Secondary endpoints were blood parameters before and after surgery. An overview of the study procedure is given in Figure 1A. Each study participant received a Somagen^®^ device performing either placebo or ELF-PEMF treatment. Treatments (7 min per day) were started one day after surgery and were repeated for the following 30 days. For the treatment, the applicator was placed over the HTO at the proximal tibia (Figure 1B).

### 2.4. Generation of ELF-PEMF

The specific ELF-PEMF used in this study, has a fundamental frequency of 16 Hz and an intensity of 6-282 µT (B field amplitude 6 mm above the applicator), which is emitted as groups of pulses (bursts) in send–pause intervals. ELF-PEMF (and placebo) were generated with the Somagen^®^ (Sachtleben GmbH, Hamburg, Germany) device for external application of the ELF-PEMF. The Somagen^®^ is a portable device certified for medical use according to European laws (CE 0482 according to 93/42/EEC Annex V, compliant with EN ISO 13485:2012 + AC:2012).

### 2.5. Randomization and Unblinding

Randomization was performed with the help of MS Excel at Sachtleben GmbH. Sachtleben GmbH generated 75 blinded treatment cards (randomized for placebo and ELF-PEMF) for the Somagen^®^ devices. This way, devices did not differ in design or handling. Neither patient nor attending physician could perceive which condition was assigned. This approach guaranteed concealment of allocation and double-blinding. Unblinding was performed after all data were collected and saved. For that reason, treatment cards were read out by a non-public software (technical control) at Sachtleben GmbH.

### 2.6. Surgery and Post-Surgical Treatment

High tibial osteotomy was performed in a slightly modified technique, according to Lobenhoffer et al. and Staubli et al. [27,28]. A vertical 6 cm medial approach (by three different surgeons) was used. The insertion of the patellar tendon and the hamstring tendons were identified. Depending on the tension of the MCL, a partial or complete release of the sMCL was performed. K-wires were inserted just above the tendon of the gracilis muscle and exactly according to the preoperative planning. The aiming point was the upper third of the fibula head. The biplanar osteotomy started with a transversal cut, which was set parallel to the lateral tibial slope with an oscillating saw (DePuy Synthes TRS, saw blade Komet—1.27 mm thickness) followed by the biplanar (ascending) cut in a 110° angle behind the insertion of the patellar tendon, and pointing towards the tibiofibular joint. The osteotomy was slowly opened with chisels and slowly spread using a spreader at the posterior border of the tibia to achieve a gap size in accordance with the preoperative digital planning tool [29]. 1.27 mm were added, due to the loss of bone using the saw blade. The alignment was confirmed intraoperatively with an alignment rod by an image intensifier to avoid huge over- or under-correction. There was no “fine-tuning” according to the alignment rod, just a final control to avoid complications. To fix the osteotomy, we used a TomoFix^©^ medial high tibial plate fixation (DePuy Synthes, Umkirch, Germany) with corresponding titanium locking screws [28]. Physiotherapy started one day post-surgery, after the removal of the drainage. Immediately after the surgery, exercises with no limitation on range of motion were allowed. No lower leg casts were used. All patients were mobilized with partial weight-bearing of 20 kg on crutches for 2 weeks. After this time, the patients could gradually increase weight-bearing. Clinical and radiological examinations were planned 6 and 12 weeks after surgery (part of the clinical follow-up) with X-rays in an anterior-posterior and lateral view, as well as a bilateral long leg view.

### 2.7. Osseous Consolidation

Bone healing of osteotomy gaps in conventional radiographs is normally observed from the lateral to the medial hinge. The hinge generates a connection between the proximal and distal part of the osteotomy and is plastically deformed when opening the osteotomy gap. An early sign of bone regeneration is the increase in bone density at the osteotomy surfaces. To quantify osseous consolidation, both the distance of the osteotomy from medial to lateral (distance A), and the part of the osteotomy gap already filled with bone (distance B), was measured. The percentage of the osteotomy gap filled with new bone (osseous consolidation) is obtained by dividing distance B by distance A (Figure 1C), as previously described [26]. Radiographs were analyzed by three independent and blinded specialists in radiology or orthopedic surgery. Multicollinearity between the three investigators revealed a *R*^2^ = 0.849 for the first X-ray and *R*^2^ = 0.762 for the second X-ray. Thus, the geometric means of these measurements were used for further evaluation.

### 2.8. Blood Parameters

Serum and plasma (EDTA plasma) blood samples were taken from the patients’ pre- and post-surgery (before discharge of the hospital) as a part of a routine blood test. Besides the routine blood cell count, serum levels of bone-specific alkaline phosphatase (BAP: osteoblast function) and tartrate-resistant acidic phosphatase (TRAP5b: osteoclast function) were determined with the help of ELISA kits (AC-20F1 and SB-TR201A—IDS, Frankfurt, Germany). Furthermore, serum levels of tumor necrosis factor alpha (TNF-α: inflammatory response), interleukin 6 (IL-6: inflammatory response), monocyte chemoattractant protein 1 (MCP-1: oxidative stress response [30]), and interleukin 13 (IL-13: regulator of hematopoiesis [31]), were determined with the help of standard ABTS ELISA kits (900-K25, 900-K16, 900-K31, and 900-K23—PeproTech, Hamburg, Germany). ELISAs were performed according to the manufacturer’s protocols.

### 2.9. Statistics

Statistical planning was performed by the Institute for Clinical Epidemiology and Applied Biometry before the study. To detect a 10% difference in means, with a common standard deviation of 12% in osseous consolidation, with the two-sided *t*-test and 80% power (α = 0.05, two-sided significance level), the required total sample size is *N* = 48 (24 patients per group). Due to possible drop-outs (discontinuation, loss to follow up, etc.), it was planned to include 75 patients in the study.

Results are expressed as scatter diagrams (mean ± 95% CI) and described as mean ± standard error of the mean (SEM). The number of biological (*N*) and technical (*n*) replicates are given in the figure legends. Normal distribution of data was not given (D’Agostino & Pearson omnibus normality test). Therefore, data sets were compared by two-tailed Mann–Whitney *U*-test. Statistical analysis was performed using the GraphPad Prism 5 software (El Camino Real, USA) and reviewed by an independent statistician. The global significance level was set to α = 0.05. As we hypothesized a stronger effect in older patients, a subgroup analysis was performed for patients ≥50 years. Additional subgroup analyses were done for sex, BMI, and wedge height.

## 3. Results

**Figure 2:**
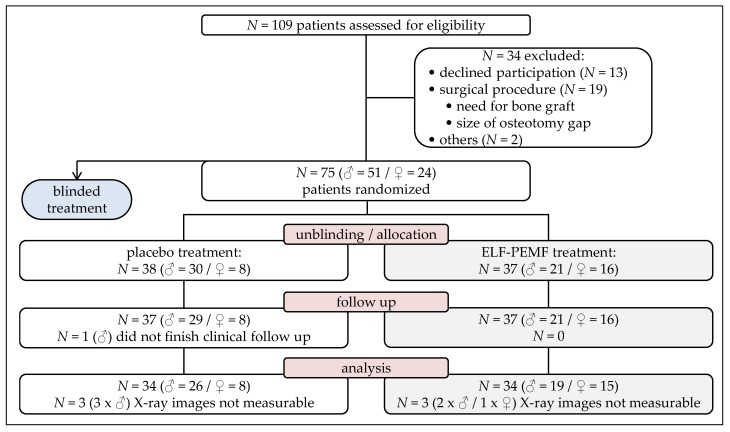
Flow diagram on patient recruitment and study population.

Between September 2015 and November 2017, 109 patients, receiving an HTO due to a varus malalignment of their mechanical axis, were informed about the study. Thirteen patients declined to participate. Nineteen had to be excluded due to the necessity of intra-surgical bone grafting or the resulting size of the opening gap. A further two patients had to be excluded for other reasons. The remaining 75 patients were randomized to ELF-PEMF or placebo treatment. Of these, one patient did not finish the post-surgical care, and was therefore rated as lost to follow-up (Figure 2).

Thus, data of 74 patients could be included in the present study. Unblinding revealed that 37 patients received ELF-PEMF treatment, and 37 patients received placebo treatment. Table 1 shows that, before surgery, both groups were comparable concerning demographics and risk factors.

Furthermore, both groups were comparable concerning laboratory parameters before surgery (Table 2). Solely, thrombocyte count was different between the placebo (241.2 ± 7.8 × 10^3^/µL) and the ELF-PEMF group (275.2 ± 10.9 × 10^3^/µL; *p* = 0.031).

### 3.1. Excellent Acceptance and Compliance of the Treatment

For the compliance check, after the 30-day application the patients’ documentation was compared with the readout of the treatment cards (technical control). Both the patients’ documentation (average: 32.0 ± 0.5 applications/patient) and the readout of the treatment cards (average: 33.6 ± 0.4 applications/patient) showed an excellent compliance among the study patients. Furthermore, post-surgical complications requiring intervention occurred only in few cases: instable hinge fracture (one in each group), infection (one in each group), compartments syndrome (one in the placebo group), and hematoma (one in the ELF-PEMF group). Of these, none could be attributed to the ELF-PEMF treatment.

### 3.2. Osseous Consolidation in the ELF-PEMF and the Placebo Group

**Figure 3:**
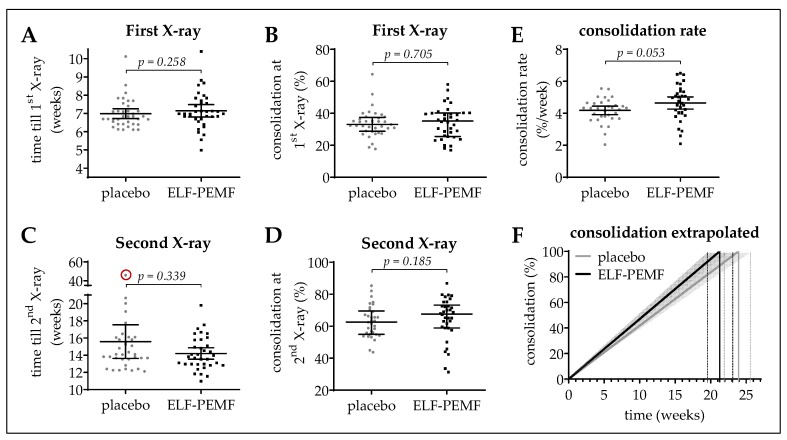
Osseous consolidation in the study cohort. (**A**,**C**) show the time from surgery till first (1st) or till second (2nd) X-ray, respectively. (**B**,**D**) show the osseous consolidation at the 1st and 2nd X-ray, respectively. Osseous consolidation is given in %. (**E**) The consolidation rate was determined as: Δ osseous consolidation (2nd X-ray—1st X-ray)/Δ time (2nd X-ray—1st X-ray). Data are represented as scatter diagrams (mean ± 95% CI). (**F**) Extrapolation of the resulting mean (line) ± SEM (surrounding area) consolidation rates of the ELF-PEMF and placebo group. Two-tailed Mann–Whitney *U*-test was used to determine *p*-values. *N* = 34 per group (geometric mean of *n* = 3).

The first X-ray (starting point) of the affected leg was performed on average 7.1 ± 0.1 weeks after surgery. The duration from surgery until first X-ray was comparable between the ELF-PEMF (7.1 ± 0.2 weeks) and the placebo group (7.0 ± 0.1 weeks; Figure 3A). Similarly, the osseous consolidation at the first X-ray did not differ between the ELF-PEMF (35.3 ± 1.7%) and the placebo group (35.6 ± 1.7%; Figure 3B).

At the second X-ray, no significant difference in osseous consolidation could be observed between the ELF-PEMF (64.7 ± 2.4%) and the placebo group (62.8 ± 1.3%; Figure 3D). However, the time from surgery until the second X-ray was, on average, 9.7 days longer in the placebo group (15.7 ± 1.0 weeks) when compared to the ELF-PEMF group (14.2 ± 0.3 weeks; Figure 3C). Due to this difference in timing, we determined the patients’ individual consolidation rates (increase in osseous consolidation between 6 weeks and 3 months after surgery in % divided by time in weeks). The mean consolidation rate in the ELF-PEMF group (4.64 ± 0.19%/week) was slightly higher than the mean consolidation rate in the placebo group (4.18 ± 0.13%/week; Figure 3E), but just missed statistical significance. The resulting consolidation rates were extrapolated in Figure 3F.

### 3.3. Early Increase in BAP Serum Levels in the ELF-PEMF Group

**Figure 4:**
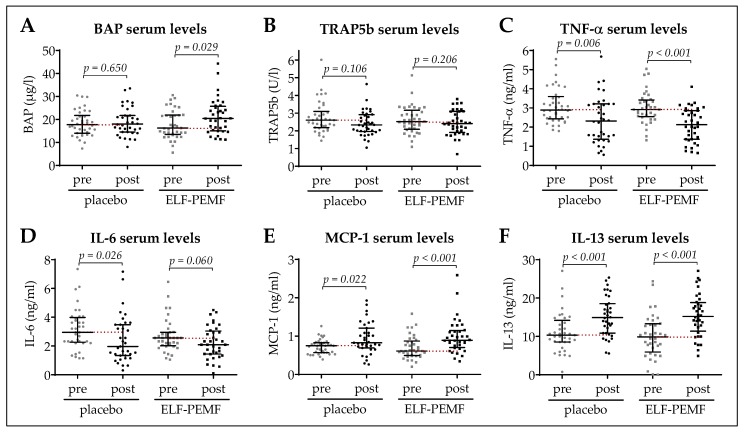
Pre- and post-surgical serum analysis. Serum levels of (**A**) bone-specific alkaline phosphatase (BAP), (**B**) tartrate-resistant acidic phosphatase (TRAP5b), (**C**) tumor necrosis factor alpha (TNF-α), (**D**) interleukin 6 (IL-6), (**E**) monocyte chemoattractant protein 1 (MCP-1), and (**F**) interleukin 13 (IL-13). Data are represented as scatter diagrams (mean ± 95% CI). Placebo: *N* = 36 and ELF-PEMF: *N* = 37 (*n* = 2). Two-tailed Mann–Whitney *U*-test was used to determine *p*-values, as indicated.

BAP, TRAP5b, TNF-α, IL-6, MCP-1, and IL-13 serum levels were determined before and 4.13 ± 0.15 days (all patients) after surgery (Figure 4A–F). The mean time between surgery and post-surgical blood sampling was comparable between both groups. BAP serum levels (osteoblast function) showed a significant increase from 17.9 ± 1.0 µg/L to 21.3 ± 1.3 µg/L in the ELF-PEMF group (*p* = 0.029), which was not observed in the placebo group (18.4 ± 0.9 µg/L to 19.1 ± 1.0 µg/L; Figure 3A). TRAP5b serum levels (osteoclast function), were not significantly different or altered in the two groups (Figure 4B). TNF-α and IL-6 serum levels decreased significantly in both the placebo and the ELF-PEMF group (Figure 4C,D). MCP-1 serum levels (oxidative stress) showed a more pronounced increase in the ELF-PEMF group when compared to the placebo group (Figure 4E). The increase in IL-13 serum levels was comparable between both groups (Figure 4F).

### 3.4. Osseous Consolidation Was Independent of Sex, BMI, or Wedge Height

**Figure 5:**
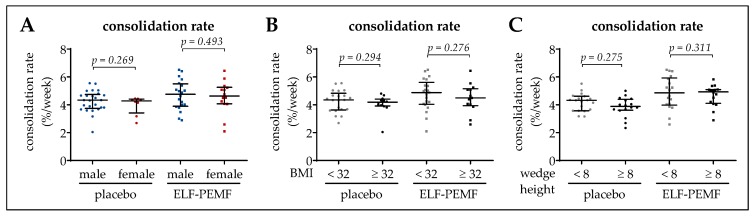
Effect of sex, BMI and wedge height on the osseous consolidation. (**A**) Sex effect on the rate of osseous consolidation. (**B**) A BMI of 32 kg/m^2^ was set as inflection point to investigate the effect of BMI on the consolidation rate. (**C**) A wedge height of 8 mm was set as inflection point to investigate the effect of the wedge height on the consolidation rate. Data are represented as scatter diagrams (mean ± 95% CI). Two-tailed Mann–Whitney *U*-test was used to determine *p*-values, as indicated. *N* = 68 data points (geometric mean of *n* = 3) were used for the individual analyses.

In order to identify factors, which might influence the ELF-PEMF therapy, subgroup analyses were performed for the effect of sex, BMI, and wedge height on the rate of osseous consolidation. However, these factors did not influence the rate of osseous consolidation (Figure 5A–C).

### 3.5. ELF-PEMF Treatment Improved Osseous Consolidation in Elderly Patients

**Figure 6:**
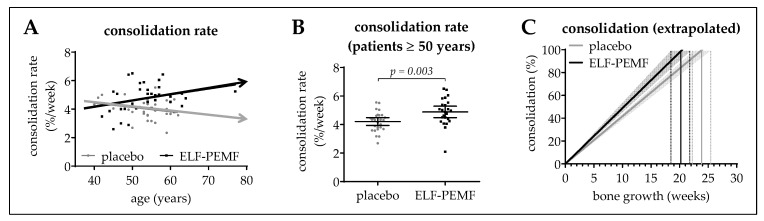
Effect of the patients’ age on the osseous consolidation. (**A**) Correlation between the osseous consolidation rate and the patients’ age. Trend-lines on the X/Y-diagram cross at an age of ~52 a. (**B**) Consolidation rate in patients ≥ 50 a (placebo: *N* = 27; ELF-PEMF: *N* = 24). Data are represented as scatter diagram (mean ± 95% CI). Two-tailed Mann–Whitney *U*-test was used to determine the *p*-value. (**C**) Extrapolation of the resulting mean (line) ± SEM (surrounding area) consolidation rates of the ELF-PEMF and placebo group.

Correlating the patients’ age with the osseous consolidation rate showed an age-dependent decrease in the placebo group (slope = −0.235 ± 0.317%/a). This effect was reversed in the ELF-PEMF group, which showed an age-dependent increase in the rate of osseous consolidation (slope = 0.417 ± 0.321%/a; Figure 6A), resulting in a significantly higher (*p* = 0.003) consolidation rate in the subgroup of elderly patients (≥50 years/*N* ≥24 per group). While the consolidation rate remained constant at 4.2 ± 0.1%/week in the placebo group, it increased to 4.9 ± 0.2%/week in the ELF-PEMF group (Figure 6B). Extrapolation of the consolidation rates is shown in Figure 6C. Information on the study cohort is given in Table 3.

## 4. Discussion

To our knowledge, this is the first double-blind, randomized, placebo-controlled, clinical trial evaluating simultaneously osseous consolidation and blood serum markers, to investigate the effects of ELF-PEMF on osseous consolidation after HTO. In our study, ELF-PEMF-treated patients showed higher consolidation rates than placebo-treated patients, with the effect being significant with increasing age. We expected a stronger effect in elderly patients (≥50 years). However, we were unsure about recruitment time, in case of restricting inclusion to elderly patients. The sample size for the group of patients aged ≥50 years was 51. Hence, this sample size would have been sufficient to base the study on patients with age ≥50 years demonstrating superiority of ELF-PEMF over placebo with 80% power. Extrapolating the obtained mean consolidation rates suggested that 100% osseous consolidation is reached 4–5 weeks faster in ELF-PEMF-treated patients than in placebo-treated patients.

The positive effects on osseous consolidation were supported by an early increase in BAP in the ELF-PEMF group, indicating an increased osteoblast function in these patients. This is in line with the preceding in vitro study, showing accelerated maturation (AP activity, matrix mineralization and expression of osteogenic transcription factors) of primary human osteoblasts exposed to the same ELF-PEMF used in this study [22]. In the in vitro studies, the ELF-PEMF applied did not enhance osteoclast viability or function [22,32]. Thus, it was not surprising that TRAP5b serum levels were not induced in ELF-PEMF-treated patients.

Some recent studies question the effect of ELF-PEMF treatment on osseous consolidation: PEMF therapy could not reduce delayed or non-union rates in acute tibial shaft fractures, which might be partly due to the small sample size used in this study [33]. Investigating PEMF therapy for osteochondral lesions of the talus, no significant differences in osseous repair or secondary functional outcomes between the placebo and the PEMF group could be found [17]. However, the investigated study cohort was, with a mean age of 32 ± 10 years in the PEMF group and 34 ± 7 years in the placebo group [17], around the maximum bone strength in life. Following our findings, ELF-PEMF effects are stronger in elderly patients. This is conceivable, as age is known to be a risk factor delaying osseous consolidation [34,35]. The studies of Hannemann et al., fortify this assumption: in a study population with a mean age of 35 years, PEMF treatment failed to improve clinical and radiological outcomes of acute scaphoid fractures [9,36]. In addition, missing placebo controls, inhomogeneity of fractures, and great variety in treatment strategies/regimes and outcome parameters, further impedes comparison of available studies, as has been summarized before [20,21].

Our preceding work, which investigated the effect of 10 different ELF-PEMFs (blinded) on primary human osteoblasts (mean donor age: 69.5 ± 8.6 a), revealed a frequency-dependent effect of the ELF-PEMF on viability and function [22]. The strongest effects were observed with the ELF-PEMF (16 Hz) used in this study. With frequencies over 33 Hz, no effect on primary human osteoblast viability and function were observed [22], this might explain the lacking effect of the PEMF (75 Hz) in the study of Reilingh et al. [17]. In another study, however, a higher rate of osseous consolidation after intramedullary nailing of tibial pseudarthrosis was shown, when patients were additionally exposed to ELF-PEMF (75 Hz) [37]. Lower frequency ELF-PEMF (15 Hz) proved to be effective in treating non-unions of long and non-long bone fractures [38]. This finding is in line with a report on earlier removal of the external fixation in distraction osteogenesis for limb lengthening in ELF-PEMF stimulated bones [39]. The above-mentioned studies investigated patients with rather poor bone metabolism. These findings support our hypothesis that ELF-PEMF therapy mainly supports osseous consolidation in patients with risk factors for pseudarthrosis or delayed union, e.g., elderly or malnourished patients, smokers, diabetics, or patients frequently consuming alcohol or using non-steroidal anti-inflammatory drugs (NSAIDS) [3,40]. Therefore, it seems reasonable to use this therapy as an adjunct for conventional therapy in patients with poor bone metabolism, like elderly, diabetics, smokers, and patients with bad bone quality (osteopenia/osteoporosis).

Furthermore, the duration and time of therapy vary strongly between the reported studies. Most studies applied their PEMFs for a minimum of 8 h per day for several months [37,38,39]. In our study, ELF-PEMF was applied 7 min per day for an average of 33.6 ± 0.4 days, which is well before the second X-ray, planned after 12 weeks, used for evaluation. Thus, it remains to be clarified whether longer and/or more treatment intervals may further improve the clinical and radiological outcome, although, it has been reported that longer exposure periods and therapy duration might reduce compliance [24].

The preceding in vitro studies identified superoxide radical and hydrogen peroxide formation by the ELF-PEMF [23], and suggested the induction of MAPKinases (especially ERK1/2 [22]) is crucial for the observed effects on human osteoblasts. This was observed rapidly after exposure to the ELF-PEMF, such that 7 min treatment intervals seemed reasonable. Indeed, patients in the ELF-PEMF group showed a rapid increase in serum levels of MCP-1, which is reported to be induced by oxidative stress [30].

TNF-α and IL-6, increased during inflammatory responses, are reported to induce bone marrow macrophages to differentiate into osteoclast-like cells with a bone-resorptive activity in a RANKL (Receptor activator for nuclear factor κB ligand)-independent manner [41,42]. Compared to the placebo group, ELF-PEMF treatment did not affect TNF-α or IL-6 serum levels in our study cohort, suggesting that no inflammatory response occurred that could negatively affect bone quality. Similarly, changes in IL-13 serum levels were comparable between ELF-PEMF and the placebo group. This is important, as IL-13 enhances the proliferation of bone marrow progenitor cells and thus regulates hematopoiesis [31].

The limitations of this study are the relatively short follow-up period and the radiological observation method. A post-surgical CT scan could not be performed, as it is not necessary for regular post-surgical care. We consider the randomized, double-blind, placebo-controlled design and the very homogeneous study population as major strengths. Moreover, the patients’ compliance in applying the ELF-PEMF therapy, and the correlating serum marker investigations, which correlate well with preceding in vitro findings, support the significance of the study. Recent studies have also suggested that ELF-PEMF treatment might be favorable for treating osteoporosis in post-menopausal women [13,43,44]. Therefore, investigating the effects of ELF-PEMF therapy on bone mineral density should be considered for further studies, especially when targeting elderly patients. Additionally, more studies on cost-effectiveness would help the physician in charge to decide about the use of ELF-PEMF treatment to support bone formation.

## 5. Summary and Conclusions

To summarize, this specific ELF-PEMF therapy showed a tendency to speed up osseous consolidation after HTO, with respect to the entire study group. The effect was significant for patients over 50 years. These results were supported by an early increase in BAP serum levels in the ELF-PEMF group. The lacking influence of the ELF-PEMF treatment on serum levels of TRAP5b, TNF-α, IL-6, and IL-13 suggests no adverse effects of the treatment. Considering our study results, ELF-PEMF treatment might be a useful approach to support osseous consolidation, especially in older patients or patients with poor bone metabolism.

## Figures and Tables

**Table 1:** Age, sex, wedge height, body mass index (BMI), comorbidities, and nutritional status (NRS) of the study cohort prior to surgery.

Item		Placebo	ELF-PEMF	*p*-Value *
Number of patients	(1)	37	37	-
Sex distribution	(♂/♀)	29/8	21/16	0.081
Age	(a)	52.9 ± 1.0	54.1 ± 1.3	0.865
BMI	(kg/m^2^)	29.9 ± 0.6	29.9 ± 1.0	0.887
Mean wedge height	(mm)	7.8 ± 1.8	7.6 ± 1.5	0.872
Number of co-morbidities	(1)	2.8 ± 0.2	2.6 ± 0.3	0.139
NRS	(1)	0.34 ± 0.09	0.43 ± 0.09	0.463

Age, BMI, mean wedge height, comorbidities, and NRS are represented as mean ± SEM (95% C.I.); * Fisher’s exact test (sex distribution) or two-tailed Mann–Whitney *U*-test.

**Table 2:** Data on full blood cell counts taken prior to surgery.

Pre-Surgical Blood Values		Placebo	ELF-PEMF	*p*-Value *
monocytes	(10^3^/µL)	0.43 ± 0.03 (0.38–0.48)	0.44 ± 0.02 (0.39–0.48)	0.791
leucocytes	(10^3^/µL)	7.3 ± 0.3 (6.7–8.0)	6.8 ± 0.3 (6.3–7.4)	0.328
lymphocytes	(10^3^/µL)	1.9 ± 0.1 (1.6–2.1)	2.0 ± 0.1 (1.7–2.3)	0.956
neutrophils	(10^3^/µL)	4.4 ± 0.3 (3.8–5.0)	4.1 ± 0.2 (3.7–4.5)	0.405
eosinophils	(10^3^/µL)	0.17 ± 0.02 (0.13–0.21)	0.2 ± 0.0 (0.1–0.2)	0.817
basophils	(10^3^/µL)	0.05 ± 0.01 (0.04–0.06)	0.05 ± 0.00 (0.04–0.06)	0.900
erythrocytes	(10^6^/µL)	4.9 ± 0.1 (4.8–5.0)	4.8 ± 0.1 (4.7–5.0)	0.646
HB	(g/dL)	15.1 ± 0.2 (14.8–15.4)	14.6 ± 0.2 (14.2–15.0)	0.061
HKT	(%)	44.2 ± 0.5 (43.3–45.2)	43.3 ± 0.6 (42.1–44.4)	0.141
MCV	(fL)	90.8 ± 0.6 (89.5–92.1)	90.0 ± 0.7 (88.5–91.5)	0.459
MCH	(pg)	31.0 ± 0.2 (30.5–31.4)	30.4 ± 0.3 (29.8–30.9)	0.203
MCHC	(g/dL)	34.2 ± 0.1 (33.9–34.4)	33.8 ± 0.1 (33.6–34.0)	0.104
thrombocytes	(10^3^/µL)	241.2 ± 7.8 (227.8–264.4)	275.2 ± 10.9 (253.1–297.4)	0.031
Quick	(%)	106.6 ± 2.6 (101.5–111.6)	112.5 ± 2.3 (107.9–117.1)	0.299
INR	(1)	0.99 ± 0.01 (0.96–1.01)	0.96 ± 0.01 (0.95–0.98)	0.309
PTT	(s)	29.1 ± 0.5 (28.0–30.1)	29.0 ± 0.5 (27.9–30.1)	0.746
CRP	(mg/L)	6.0 ± 2.3 (1.5–10.5)	7.2 ± 2.4 (2.4–12.1)	0.668
creatinine	(mg/dL)	0.96 ± 0.03 (0.90–1.02)	0.91 ± 0.03 (0.85–0.97)	0.310
sodium	(nmol/L)	141.6 ± 0.3 (141.0–142.3)	141.0 ± 0.4 (140.2–141.8)	0.227
potassium	(nmol/L)	4.2 ± 0.1 (4.1–4.4)	4.3 ± 0.1 (4.1–4.4)	0.623
GOT	(U/L)	24.3 ± 1.7 (20.5–27.5)	23.4 ± 1.4 (20.6–26.3)	0.946
g-GT	(U/L)	44.6 ± 11.0 (22.0–65.5)	31.6 ± 5.7 (20.0–43.1)	0.572
glucose	(mg/dL)	110.8 ± 4.6 (101.3–119.7)	105.2 ± 4.0 (97.0–113.5)	0.605

Data are represented as mean ± SEM (95% confidence interval); * two-tailed Mann–Whitney *U*-test.

**Table 3:** Pre-surgical age, sex, wedge height, body mass index (BMI), comorbidities, and nutritional status (NRS) of the patients ≥50 years in our study cohort.

			Placebo	ELF-PEMF	*p*-Value *
Number of patients		(1)	27	24	-
Sex distribution		(♂/♀)	20/7	13/11	0.156
Age	all	(a)	55.6 ± 0.7	57.5 ± 1.3	0.398
	♂	(a)	56.3 ± 0.8	55.5 ± 1.1	0.612
	♀	(a)	53.3 ± 0.7	58.6 ± 2.2	0.056
BMI		(kg/m^2^)	29.6 ± 0.7	29.5 ± 1.1	0.578
Mean wedge height		(mm)	7.9 ± 0.3	7.8 ± 0.3	0.834
Number of co-morbidities		(1)	2.9 ± 0.2	2.8 ± 0.4	0.655
NRS		(1)	0.38 ± 0.10	0.29 ± 0.10	0.513

Age, BMI, mean wedge height, comorbidities and NRS are represented as mean ± SEM (95% C.I.); * Fisher’s exact test (sex distribution) or two-tailed Mann–Whitney *U*-test.

## Abbreviations

BAP	bone specific alkaline phosphatase
BMI	body mass index
ELF-PEMF	extremely low frequency pulsed electromagnetic field
ELISA	enzyme-linked immunosorbent assay
HTO	high tibial osteotomy
IL-13	interleukin 13
IL-6	interleukin 6
MCP-1	monocyte chemoattractant protein 1
NRS	nutritional risk screening 2002
TNF-α	tumor necrosis factor alpha
TRAP5b	tartrate resistant acidic phosphatase
